# 

**Published:** 1898-01

**Authors:** 


### MEETING OF THE SOUTHERN SECTION OF THE AMERICAN LARYNGOLOGICAL, RHINOLOGICAL AND OTOLOGICAL SOCIETY.

We wish to call attention to the meeting of the above society which will convene in Atlanta about the 28th of March. An invitation is extended to all physicians, but especially to those who do ear, nose, and throat work. This is a new society, but one which is doing very progressive work. Dr. A. W. Calhoun is Chairman of the Southern Section and Dr. Dunbar Roy is Secretary.

The Chairman extends an invitation to doctors engaged in this special line of work all over the South to attend this meeting and contribute a paper.

